# The influence of the fourth industrial revolution in teaching and learning: the COVID-19 context

**DOI:** 10.12688/f1000research.131618.2

**Published:** 2024-06-27

**Authors:** Awelani V. Mudau, Lettah Sikhosana

**Affiliations:** 1 Department of Science and Technology Education, University of South Africa, Pretoria, Gauteng, South Africa

**Keywords:** Fourth Industrial Revolution, School Management Teams, Integration, Blended learning.

## Abstract

**Background:**

The aim of this paper was to explore how the Fourth Industrial Revolution shapes teaching and learning during COVID-19 in some of the schools located in the Gauteng and Mpumalanga provinces, South Africa. This paper employed qualitative interpretative multiple case study design. Purposive sampling was used to sample our participants.

**Methods:**

We selected four teachers who separately taught at the early childhood development stage, intermediate phase, senior phase, and further education and training phases. Data was collected telephonically through semi-structured interviews with teachers from Limpopo, Gauteng and Mpumalanga provinces of South Africa. Data was collected from their experiences of COVID-19 from 2019 to 2021. Collected data was analysed using a typology approach whereby themes that derived from the literature reviewed and research questions were used.

**Results:**

We inferred from the results of the paper that teachers had challenges with teaching and availability of learning resources, such as limited access to internet and socio-economic backgrounds, which hindered the integration of the Fourth Industrial Revolution in the teaching and learning process. There were also challenges related to teachers’ background on the usage of the Fourth Industrial Revolution and the lack of school management teams support. The paper revealed that the integration of the Fourth Industrial Revolution in teaching and learning was affected negatively by the existence of the above-mentioned challenges, which needs to be addressed.

**Conclusions:**

Therefore, we recommended that the government and stakeholders within the education sector provide resources such as smart-boards, computers, and network access in schools lacking such facilities, as well as providing professional development interventions and training teachers to have an in-depth understanding of the Fourth Industrial Revolution within the teaching and learning context.

## Introduction

The effect on the COVID-19 pandemic has exposed a number of imbalances in the South African education system. COVID-19 also showed the impediment of teacher and learner knowledge in digital learning and teaching in this Fourth Industrial Revolution (4IR) era as most of the learners were unable to use these technological devices. Whereas teachers also were afraid to explore the possibilities of teaching using the technology just like some of their learners. Davis (2016) defines 4IR as “the fusion of technologies that is blurring the lines between the physical, digital, and biological worlds”. The pedagogical knowledge during teaching practice becomes the contested phenomenon in the era of the 4IR.

Inadequate technological or digital knowledge negatively affects the existing South African Curriculum and Assessment Policy Statement (CAPS) subjects, despite the attempt at introducing the evolution of 4IR as part of the school curriculums ( Mudau and Sikhosana, 2024). The stakes of knowledge possession in education during the 4IR era is high and is the prevailing phenomenon for an effective digital teaching during Education and Information Technologies ( Grossman, 2018). Moreover, Mpungose (2016) agrees with Nkambule and Mukeredzi (2017) that having adequate technological teaching knowledge in the field of education leads to advanced ways of knowing the subject content, pedagogical strategies, and digital resources that are appropriate and needed.

One of the key rules of the COVID-19 pandemic, which is the social distancing, has compelled the teaching process to take a form of a virtual classroom or online interactive forum, or digital ways that includes; Microsoft teams teaching, google classroom teaching, telegram teaching, WhatsApp teaching, zoom teaching, and hub teaching ( Mpungose, 2019). The hub digital system is when one of the classroom at school is used as a center to connect digitally, the other classrooms where learners are seated in view of observing all the covid-19 rules to combat the spread of the corona virus.

Currently, there is no available model or empirically based report that shows positive correlation between closure of schools and curbing of the spread of COVID-19 ( Viner *et al.,* 2020). However, the recent developments in technology 4IR demand more intimate technological knowledge about more advanced digital technologies, such as the internet and digital video ( Schäfer, 2018). Schwab (2016) resists that 4IR is influenced by a much more advanced knowledge on a universal and internet, by smaller and more powerful sensors that have become cheaper, and by artificial intelligence and machine learning. As a result, this paper explored how the 4IR shaped teaching and learning during the COVID-19 pandemic in some of the schools located in Gauteng and Mpumalanga province, South Africa.

### Literature review

COVID-19 is a contagious disease caused by a new virus known as SARS-CoV-2 ( World Health Organisation [WHO], 2020). This novel disease was originally known in China in Wuhan city in 2019. Mahaye (2020) indicates that South Africa has its first confirmed case of COVID-19 on 5 March 2020 and, within a month, the total of infected cases increased to 1,585. As of 23 April 2020, the number of confirmed cases in South Africa increased to 3,953 with 75 related deaths. COVID-19 is spread from one person to another through respiratory droplets that are expelled when an individual with the disease sneezes, coughs, or talks ( Centers for Disease Control and Prevention [CDC], 2020).

Thus, medical specialists suggested social distancing as a means of containing the transmission of the dreaded virus ( CDC, 2020). Mahaye (2020) states that the rapid spread of the pandemic in several countries of the world led to the temporary closure of schools at all levels of learning. The global statistics has it that above1.6 billion of youngsters have been out of school due to the abrupt and short-term closure of institutions ( World Bank, 2020). The aftermath of the school closure propelled governments across the world to embrace distance learning to curtail the spread of the virus and enable home learning despite the virulent pandemic.

Thus, the World Bank (2020) is working relentlessly to support Ministries of Education to offer online learning to various institutions of learning during the ravaging pandemic. All schools in South Africa were temporarily closed down on 27 July 2020 during the pandemic rise and alternative methods of teaching such as blended learning was effectively introduced to the school systems to allow learners’ access to education during the lockdown period ( Mahaye, 2020).

### The Fourth Industrial Revolution (4IR)

The World Economy Forum (2018) indicates that 4IR connotes technological innovation that shapes the boundaries between physical, biological, and digital worlds. Kayembe and Nel (2019) also state that 4IR is based on the present digital revolution that leads to the creation of fresh opportunities and possibilities for society. Erboz (2017:2) contends that the components of 4IR include the Internet of Things (IoT), Cyber-Physical Systems, Internet of Services, and Smart Factory. Other components are artificial intelligence, three-dimensional (3D) printing, robotics, block chain technology, cryptocurrency, quantum computing, nanotechnology, and bioengineering. Universally, cloud-based platforms like Zoom, Google Hangouts, and WebEx, are useful for teachers and learners to complete their tasks from home during the pandemic. Beetham and Sharpe (2013) indicate that digital technology promotes interactions between teachers and learners thereby transforming the teaching and learning activities. However, Rashid and Asghar (2016) found that there is insignificant impact of digital technologies on learners’ academic performance.

Kayembe and Nel (2019) found that the education system in South Africa is facing some challenges during 4IR implementation. The challenges are inadequate funding, infrastructure, and adequate skills needed for 4IR implementation. The teachers’ challenges are in the areas of pedagogical adaptation, teacher development and infrastructure for technological innovation ( Kayembe and Nel, 2019). The 4IR provides the following opportunities such as:

• Provision of an environment of creativity and innovations ( Kayembe and Nel, 2019).
• The application tends to solve social exclusion issues ( Chetty and Pather, 2015:5) on how the gaps between the rich and poor can be closed as well as among the racial differences.
• The 4IR provides opportunities for the education systems to collaborate with government and private establishments ( Kayembe and Nel, 2019). Thus, based on the opportunities, the 4IR therefore, requires schools to prepare learners effectively with the necessary tools for innovation to solve immediate and future challenges in society.

### Effects of COVID-19 pandemic in the teaching and learning process

The COVID-19 pandemic led to temporary school closure that drastically affected the academic calendar ( Mhlanga and Moloi, 2020). In order to minimise the rate of academic disruptions by the nationwide lockdown, several institutions of learning embark on online programmes ( Mhlanga and Moloi, 2020). Before the pandemic, not all teachers in South Africa had the required training in line with the new technology to support blended or online learning ( The Conversion, 2020). The temporary closure of schools by the Ministry of Basic Education compelled teachers to adjust to the new technology to forestall any impediment to learning activities ( Jantjies, 2020). The teachers in South African schools had to provide adequate support to learners using online resources and face-to-face discussions on platforms like Zoom, WhatsApp, and Google apps for video calls ( Jantjies, 2020). The teachers had to supplement learning by the use of educative programmes on Radio and television ( Kuwonu, 2020). Before the pandemic, the focus of South Africa was on the digital revolution in the 4IR, thus, teachers play a leading role in digital skills progress and sustainability ( Jantjies, 2020).

### Blended learning approach during the COVID-19 pandemic

For some years now, teachers and studies have suggested blended learning for future learning ( Best, 2020). The school closure caused by the pandemic enabled the concept of blended learning to be placed into practice ( Best, 2020). Mahaye (2020) defines blended learning as a technology-based teaching system that integrate a traditional teaching method with an online learning method. Also, Best (2020) defines blended teaching as an instructional strategy that uses digital approaches in combination with conventional practice in the classroom. In this concept, digital and conventional face-face teaching may support depending on the timetable. In this case, learners might take one class in the classroom and another class entirely online.

The blended learning normally occurs in tertiary institutions. This concept can be defined as the combination of the traditional and online learning methods ( Dziuban *et al.,* 2018; Hrastinski, 2019). Its use involves learners and teachers within the classroom where face-to-face instruction is carried out. Besides, blended learning is described as the total means of teaching and learning that involve traditional face-to-face classroom methods of instruction with online learning ( Siemens, Gasevic, and Dawson, 2015). The recent lockdown has propelled teachers to implement online, blended learning teaching practices. Oliver (2020) reports that almost 10% of homes in South Africa have access to an internet connection. Limited schools implemented blended learning before lockdown and few schools adopted it in the lockdown period. Accordingly, schools with inadequate resources were redundant.

### Research questions

This paper was guided by the need to explore how the 4IR shaped teaching and learning during the COVID-19 pandemic in some of the schools located in Gauteng and Mpumalanga province, South Africa.

The following are the research questions that guided this paper:

1. How does the teacher understanding of 4IR and COVID-19 shape the integration of 4IR in teaching and learning?
2. What was the role of school management teams in the integration of 4IR in teaching and learning during COVID-19 pandemic?
3. What are the challenges and opportunities provided by the 4IR during the COVID-19 within the context of education?

## Methods

### Ethical statement

The Unisa College of Education ethics review committee approved this study (Ref: 2021/06/09/51994186/17/AM). Written informed consent was obtained from participants prior to data collection. The records are available from the authors.

### Theoretical framework

The theoretical framework for this paper was based on constructivism theory. According to Vygotsky, social constructivism propounds that people play an active role in creating their knowledge ( Schreiber and Valle, 2013). Vygotsky’s theory is based on the social construct that learning takes place in social groups ( Schreiber and Valle, 2013). He established a learning model that enables the teachers to be very active so that they will lead the learners to develop their understanding in the way they think.

Constructivism theory is a vital theory that is used in the education system. Constructivism theory was used to guide this paper as it involved the integration of 4IR in the teaching and learning. Constructivism theory has assisted the researchers in exploring teachers’ understanding of 4IR, role of SMT in the integration of 4IR as well as the challenges and opportunities in the integration of 4IR in the teaching and learning. Constructivism believes that learners should be actively involved in learning while the teachers’ role is to assist the learners in their learning activities ( Williams, 2018).

In this paper, the constructivist approach was utilised in online teaching and learning activities during the COVID-19 pandemic since access to face-to-face traditional teaching methods was not possible. These teaching and learning activities involved using mobile phones and laptops to access Google classrooms, Zoom, Microsoft Teams, and WhatsApp applications. These applications enable teachers and learners to engage themselves in knowledge construction. By using constructivism theory, the researchers were able to evaluate whether the teachers do have appropriate understanding in support of the teaching and learning through 4IR. Hence, teachers with appropriate knowledge supporting integration of 4IR understood that 4IR comprises of technology, internet and electronic devices.

In this context (4IR), teachers and learners were expected to be actively involved in teaching and learning using technology. According to Kaur (2017) in constructivist perspectives, teachers play the role of a facilitator by guiding and motivating the learners. As a result, for teachers to be able to facilitate their role in the teaching and learning through integration of 4IR, support from district officials and SMT will be required in order for learners to receive quality education. Kaur (2017) reported that teachers ensure a conducive learning environment that will enable the learners to become effective thinkers. Nevertheless, effective teaching and learning through integration of 4IR requires adequate teaching and learning resources such as smartboards, computers as well as internet access.

### Research design and methodology

This paper followed a qualitative interpretative multiple case studies design. We employed a case study design in order to provide rich descriptions of how 4IR shaped the teaching practices of teachers during the COVID-19 pandemic. We considered a case study design to be appropriate for this paper as it enabled us to gain concrete, contextual, in-depth knowledge about the phenomenon under exploration ( McCombes, 2019). In addition, the paper also focused on the teachers’ knowledge about 4IR and the challenges that teachers’ experienced when teaching during the COVID-19 pandemic in their school setting. Moreover it was plausible to use constructivism theory and in particular social constructivism as the teachers who were our case; information was sourced from them in their natural setting (that is at their schools) and they assisted the researchers to construct the data.

### Sample

The sample of this paper consisted of four teachers; two teachers taught in schools located in the Gauteng province, one teacher taught in a school located in Limpopo province and one teacher taught in the school located in Mpumalanga province. The participants were identified purposefully as they taught in different school context, in an essence such that their contexts ranged from technical (the school offered technical subjects), independent (the school was a privately owned), and government schools which we used as our selection criteria. The participants were contacted telephonically as they are our acquaintances to participate in this paper. By using a purposeful sampling, the researchers managed to include four cases according to relevant criteria, such as teachers offering teaching in any phase of school located in Gauteng, Limpopo and Mpumalanga province. Although the sample size is small, in qualitative research it is not foreign. The advantage is that it allows the in-depth analysis of the case studies. According to Crossman and Nicki (2020), purposive sampling in qualitative research is a non-probability sampling method, which researchers selected, based on the population characteristics and the aim of this paper. The selected participants had assisted the researchers in answering the proposed research questions and achieving the aim of this paper.

### Participants

The four teachers that participated in this paper were three females and one male. We used keywords and symbols for each participant. The four participants were referred to as P1/F/46/TS, P2/M/40/TS, P3/F/26/PS, and P4/F/25/GS. These symbols represent participant number, gender, age, and the nature of the school. This was also to ensure anonymity. The first two participants who were referred to as P1/F/46/TS and P2/M/40/TS were based in Limpopo province but they are currently working in Gauteng province. Three participants were teaching at the Senior (senior phase starts at grade 7 until grade 9) and FET phase (starts at grade 10 up to grade 12) whereas one participant was teaching foundation phase (starts at grade R until grade 3) and all participants were qualified to teach the subjects they were currently teaching. Moreover, the participants have different teaching experience. Table 1, below, summarises participants’ demographic details:

**Table 1.** Demographic details of participants.

Participants	P1/F/46/TS	P2/M/40/TS	P3/F/26/PS	P4/F/25/GS
Gender	Female	Male	Female	Female
Age	46	40	26	25
Nature of school	Technical	Technical	Private	Public
Qualification(s)	Bachelor of Education Honours Bachelor of Education	Bachelor of Education	Bachelor of Education Honours Bachelor of Education Master of Education	Bachelor of Education Honours Bachelor of Education
Subject(s) taught	Social sciences Life orientation	Mathematics Technical Mathematics	Mathematics	English Mathematical literacy
Grade(s) currently teaching	9-10	10-12	R (early childhood)	10-12
Teaching experience	10 Years	15 Years	3 Years	1 Year
Province	Limpopo	Limpopo	Gauteng	Mpumalanga

### Data collection instrument and procedure

Data of this paper was collected telephonically using a semi-structured interviews tool. The entire interview with each participant was in English and was audio-recorded. The rationale for this data collection method was for convenience purposes as the telephonic interviews offered an opportunity to probe and the participants were at their schools. The interviews were conducted in October to November 2021. Participants’ permission was requested by researchers prior to interview in order to ensure reliability and to avoid a mix-up of data collected during the process of analysing data. The semi-structured interview is a qualitative data collection technique where researchers prepared questions to ask the participants in advance. Additionally, in a semi-structured interview, interviewers do not necessarily follow a formalised list of questions but ask open-ended questions which allow for discussions with the interviewee ( Doyle, 2017).

The interview tool was used to collect data based on teachers’ knowledge about the 4IR in education, teacher understanding of COVID-19 pandemic, teaching and learning experience during pandemic, challenges and opportunities in integrating 4IR in teaching and learning. The recording device, i.e., audio recorder was used to capture the data of the entire interview process and researchers ensured that the participants were asked the same open questions. We developed the tool based on our research questions and the context of the study. The questions were validated by the authors. We piloted them with one of the teachers who was not part of the research.

### Rigour

To ensure that the study is trustworthy that is it is credible, transferable and dependable we ensured that the instruments are valid ( Leung, 2015). This was achieved through a pilot study which was conducted with one teacher who was not part of the main study. What we found from this pilot study was that some of the questions were not clear in terms of the language and content and we had to change them. Again the interview was too long and as we were using telephone interviews we had to reduce the questions to avoid being cut off.

### Data analysis

The data attained from four cases were analysed and interpreted separately. Researchers transcribed audio-recorded semi-structure interviews *verbatim*, to a word document. After transcribing data from the audio-recordings to word documents, the researchers replayed the audio in order to check if the words transcribed corresponded with what was on the audio thereafter they were destroyed. Moreover, researchers did not correct any grammatical errors of the participants. This was done to ensure that data collected was presented accordingly and did not lose its original meaning. Hence, the data that the researchers transcribed was communicated to relevant participants before being considered as final product.

The data collected was presented in the form of case studies i.e., case 1, case 2, case 3, and case 4. The data of this paper was analysed using Data Analysis Scheme (DAS), which was developed form the literature review, research questions and personal experiences. The data was analysed using a typology approach whereby themes that derived from the literature reviewed and research questions were used. The following are the themes derived from the literature reviewed and research questions.

➢ Understanding of 4IR and coronavirus pandemic (COVID-19) by teachers (it was necessary to understand the teachers’ perspectives of what 4IR is as well as the coronavirus pandemic as it was not going to do justice to the study to simple found out how technology was integrated without comprehending their perspectives)
➢ The role of the School Management Team (SMT) during the COVID-19 pandemic within 4IR context (in integrating technology it was important for the researchers to find out the nature of the role of the school management teams during the coronavirus pandemic)
➢ The challenges provided by the COVID-19 within the 4IR context of education (this focus on what is it that was hindering them when using or wanting to use technology)
➢ The opportunities provided by the COVID-19 within the 4IR context of education ( this focused on what opportunities were there for the teachers to integrate the 4IR)

Additionally, only data related to themes suggested for this paper were considered and assisted researchers in responding to the research questions and fulfilling the aim of this paper.

## Results

The results of each case were presented separately as a single case as our intention was not to conduct comparative research but to have an in-depth understanding of each case within their own context ( Mudau, 2023a, 2023d). We used the following keywords and symbols to present cases of each participant:

Participant 1/Female/46/technical school= **P1/F/46/TS**

Participant 2/Male/40/technical school= **P2/M/40/TS**

Participant 3/Female/26/Private school= **P3/F/26/PS**

Participant 4/Female/25/Government school= **P4/F/25/GS**

The following became our themes:

• Understanding of the 4IR and coronavirus pandemic (COVID-19) by teachers.
• The role of School Management Team (SMT) during the COVID-19 pandemic in the 4IR within the context of education.
• The challenges provided by COVID-19 within the 4IR context of education and the opportunities provided by the COVID-19 pandemic within the 4IR context of education.
• The opportunities provided by the COVID-19 within the 4IR context of education (this focused on what opportunities were there for the teachers to integrate the 4IR).

### Case 1: Participant 1/Female/46/Technical School= P1/F/46/TS

*Theme 1: Understanding of 4IR and coronavirus pandemic (COVID-19) by teachers*

For the purpose of this paper, we had to ask the teacher about her understanding of 4IR and COVID-19 within the context of the teaching and learning process. During the interview process, we noted that the teacher had an idea of what 4IR entails, as she was able to highlight some of the aspects that enables the integration of 4IR. She indicated that 4IR entails technology, internet, and electronic devices. This was evident in the statement from the interview below:

“Eh according to my understanding Fourth industrial revolution concerning teaching and learning. Is when the learners are learning through eh technology or internet or electronic devices that’s my opinion.” **P1/F/46/TS.**

We had to ask further as to what she understands about the ‘4IR’ abbreviation. She indicated that 4IR stands for “Industrial Revolution” which was incorrect in this context. This led us to probe about her understanding of COVID-19 as this shaped how 4IR is integrated in teaching and learning. However, the teacher could not explain what COVID-19 is but she was able to describe some of the COVID-19 regulations that she had to adhere too, such as using hand sanitisers and maintaining social distancing in her classroom. She indicated that:

“When I entered the classroom I had to sanitise I had to do anything that can be done eh following the protocol that they have given us and the learners were coming in twenties eh they were only 20 in the classroom.” **P1/F/46/TS.**

*Theme 2: The role of the School Management Team (SMT) during the COVID-19 pandemic within 4IR context*

The SMT plays an important role in ensuring that there is quality of teaching and learning in school. As a result, we had to find out the role that was played by the SMT during the COVID-19 pandemic within the 4IR context. The teacher revealed that the SMT was not fully involved in incorporating 4IR during COVID-19 pandemic. However, the district officials introduced the 4IR initiatives at the school with the aim of teaching teachers how to use 4IR tools such as “Zoom”. Nonetheless, they failed to complete the training task. This was evident in the statement from the interview below:

“Eh those people who were from the district they came and they said that they are going to train us for teams those zoom teams and what what but they failed because they only let us eh login only but they didn’t train us how to invite learners how to teach learners through those zoom teachings.” **P1/F/46/TS.**

It was apparent that the lack of consistency and commitment from the district officials was one of the barriers that hindered the integration of 4IR, as they did not train the teachers on how to use “Zoom” as one of the 4IR tools.

*Theme 3: The challenges provided by the COVID-19 within the 4IR context of education*

We had to probe further on other challenges that the teacher encountered when she had to integrate 4IR during the COVID-19 pandemic. Based on our findings, the teacher encountered numerous challenges to an extent that she ended up classifying herself as a “Born before Technology (BBT)” teacher. This was a challenge because she created a negative viewpoint that older teachers are not interested in integrating 4IR, as they do not have a background of using technology or adequate knowledge and skills of 4IR. She also indicated that she did not integrate 4IR during the COVID-19 pandemic as she is teaching senior phase. She asserted that:

“Eh no unfortunately no I was using chalkboard and the my chalk just because I am teaching GET or eh GET (General Education and Training phase) then which is eh senior phase which is grade 8 and 9.” **P1/F/46/TS.**

The teacher pointed out that the COVID-19 pandemic brought more challenges in the classroom, in relation to the matters of social distancing because learners were coming to school in groups and rotating. As a result, some groups would participate actively while other groups would not do that during her lessons. This was evident in the statement from the interview below:

“So eh the problem was the learners were coming in twenties eh they were only 20 in the classroom while back then before COVID-19 in the classroom they were eh 40 or 30 something. So those 20 groups of people they were concentrating and the other group were not concentrating just because eh they were rotating according to the days that they were given too yah.” **P1/F/46/TS.**

Furthermore, inadequate teaching and learning resources were a contributing factor to some of these challenges. The teacher indicated that they do have access to internet, but the computer laboratories are no longer useful since the COVID-19 pandemic. When asked if they all have their own personal laptops, she indicated that the Department of Basic Education (DBE) only offered the departmental laptops to Further Education and Training (FET) teachers in the school. This was evident in the statement below:

**“**Not all of us, those who are teaching eh senior phase they didn’t get them from the department but they are having their own personal laptops. Those who are teaching FET phase each and everyone have departmental laptops.” **P1/F/46/TS.**

Socio economic background was also a challenge that hindered the incorporation of some 4IR tools such as the creation of a WhatsApp group for the purposes of teaching and learning during the COVID-19 pandemic. We had to ask the teacher if her learners had access to smart phones. She indicated that not all of them have smart phones. As a result, it makes it difficult for her to have a WhatsApp group. This was evident in the statement below:

**“**No. I don’t have any WhatsApp group for my learners. Eh I think learners with WhatsApp groups are grade 12s only but not grade 11, 10, 9, 8 No they do not have but only Grade 12.” **P1/F/46/TS.**

As the teacher had already mentioned that she did not integrate 4IR during the COVID-19 pandemic, we had to probe further whether there were other teachers that did or did not integrate 4IR in teaching and learning during the COVID-19 pandemic. We were also interested in finding out if other teachers used tools that are aligned to 4IR such as “Zoom or Microsoft Teams” or not. The teacher asserted that:

“Eh in my school there are those subjects whereby the learners were taught through smartboards eh physical science, technical science, math’s and technical math’s the other subjects they were using chalkboards but even the mechanicals technology were using the smartboards but others were using eh chalkboard only I don’t think they were incorporating industrial revolution in it.” **P1/F/46/TS.**

Based on the above statement, it was evident that the incorporation of 4IR during COVID-19 was a personal choice as there were teachers that incorporated it while others did not.

*Theme 4: The opportunities provided by COVID-19 within the 4IR context of education*

The teacher has hope that 4IR can bring about opportunities that can make teaching and learning more effective. This was evident in the statement below:

**“**Eh I think in order to make this teaching and learning effective eh I think we can involve learner through 4IR but because it is because this learners are interactive between their cell phones mostly they are interacted in electronic devices. But, if I can put them inside the classroom, teach them with chalkboard, and chalk ah they don’t even care they are not interested anymore but if we can eh get this eh training and involve learners through electronic devices. I think it can be effective enough just because they are interested in electronic devices and their cell phones, that’s my opinion.” **P1/F/46/TS.**

### Case 2: Participant 2/Male/40/technical school = P2/M/40/TS

*Theme 1: Understanding of 4IR and coronavirus pandemic (COVID-19) by teachers.*

During the interview process, we asked the teacher what he comprehends about 4IR within the context of education. He described 4IR as:

“Eh I’m likely to be short in terms of Fourth Industrial Revolution is when we look into the system that is used for teaching and learning. Of which you will find out that there’s no longer a direct contact of the teacher and the learner. So the system that is used is part of the Forth Industrial Revolution.”

**P2/M/40/TS.**

The teacher displayed inadequate knowledge of what 4IR is, as he was of the impression that 4IR focuses on the system that limits direct contact between the teacher and the learner which, in this context, was a misconception. Furthermore, he explained that COVID-19 is a virus that spreads and affects the whole world. His understanding of COVID-19 was limited. This is evident in the statement from the interview below:

“Eh the COVID-19 pandemic is when we talk about the spreading of the virus that affects the eh the whole country or the whole world.” **P2/M/40/TS.**

*Theme 2: The role of the School Management Team (SMT) during COVID-19 pandemic within 4IR context*

We had to find out more on the roles that the SMT played during the COVID-19 pandemic towards the integration of 4IR in teaching and learning. The teacher revealed that the SMT was disorganised when it comes to the incorporation of 4IR in teaching and learning. He asserted that:

“When it comes in, we had to be on lockdown for a longer period and we couldn’t set things into place. We didn’t have contacts or the devices, we didn’t train the learners how to use the devices, we didn’t have any preparations. So during that time teaching and learning was stopped in term 1. We were focusing on direct contact. So, when learners were coming back in July, a lot was wasted. Time was no longer there we had to train the learners. That process which was supposed to be prepared before so that during from March to July we were going to be able to use that teaching and learning process, but we didn’t manage to do it because we didn’t prepare on time. So the teaching and learning was very affected.” **P2/M/40/TS.**

It was evident that the SMT played a minimal role in ensuring that the teaching and learning was not affected. There was nothing planned or prepared to continue with the lessons and this affected the process of teaching as time was also wasted.

*Theme 3: The challenges provided by the COVID-19 within the 4IR context of education*

There were also challenges when he had to integrate 4IR in teaching and learning. The teacher indicated those challenges in the statements from the interviews below:

“The challenge that we had was that majority of learners do not have access to internet. Especially on our high school learners, they do not have data. Eh not all the learners have cell phones. In terms of integrating the learners that I was working with, the majority of them, they were able to do it on their own. But with other I had to make a phone call to them explaining what they need to do. Because if I can send them a text via WhatsApp and say press this and this they are failing to follow.” **P2/M/40/TS.**

The teacher revealed that not all learners have smart phones, some do not have access to internet and, even if he can incorporate 4IR in teaching and learning, it was not effective. This is so because other learners were unable to do the work given to them. As a result, the teacher will have to spend more time in using 4IR tools such as WhatsApp and phone calls to try and explain the work given to learners individually on what needs to be done and this time consuming. When using 4IR tools such as Zoom and WhatsApp, he experienced the following challenges:

“So the challenge I was only having was the internet connection because if was using the zoom some were not going to be able to access zoom the time I’m presenting it. In terms of the WhatsApp, issue even if it’s the WhatsApp for the parent the parent will be at work after work.” **P2/M/40/TS.**

Another challenge he encountered was that some of his colleagues were reluctant in teaching online as it was based on personal interest, which led them to be resistant to change. This was evident in the statement below:

“Some of the people are afraid they got this fear of attempting things that they never done before so they prefer to do the old teaching. So what I think is better is to integrate bit by bit through the training.” **P2/M/40/TS.**

*Theme 4: The opportunities provided by the COVID-19 within the 4IR context of education*

The school itself had enough resources that can be used to incorporate 4IR during the pandemic but those resources were not used effectively. For instance, he revealed that the school has access to the following resources that were provided by the DBE which learners had an opportunity to access them:

“The school it got eh tablets, the school also have the computer lab, the school also have the internet, and the whole school has Wi-Fi. So once learners are at school, they have access to internet.” **P2/M/40/TS.**

We had to find out about the opportunities that led to the incorporation of 4IR during the COVID-19 pandemic. The teacher indicated that he did incorporate 4IR in teaching and learning during this pandemic. He incorporated 4IR by the doing the following:

“I implemented it by the means of using internet, there is other website where I log in as a teacher then I send in the question paper. The Grade 12 were doing online and other grades (8-9) were using WhatsApp.” **P2/M/40/TS.**

Furthermore, he also used 4IR tools such as Zoom, YouTube, and Microsoft Teams but his preference was WhatsApp as it was conducive for his lessons and consumes less data. This was evident in the statement below:

“Eh the platform that I prefer which is much quicker is WhatsApp because this one for zoom can only assist if there is something I want to introduce to them that they can’t be able to understand through WhatsApp. But the medium that I prefer is WhatsApp it’s the one that I was using the most and in terms of them to complete the task.” **P2/M/40/TS.**

### Case 3: Participant 3/Female/26/Private school= P3/F/26/PS

*Theme 1: Understanding of 4IR and coronavirus pandemic (COVID-19) by teachers*

The teacher’s understanding of what 4IR was in teaching and learning context was limited as she indicated that 4IR was introduced with a motive of making life, teaching and learning easier. However, she did not elaborate further about those aspects that are capable of making teaching and learning accessible. This was evident in the statement from the interview below:

“What I understand about 4IR in teaching and learning is that it’s not reflecting the teacher but for me is making life more easier and making a teacher and the learner to teach in a new way because we are in a globalised world. So it makes it easier to make information accessible for teacher and learner in terms of teaching, as they really don’t have to be face to face in order for teaching and learning to take place. So it makes teaching easy and accessible **.” P3/F/26/PS.**

The teacher showed that she had an in-depth understanding of the COVID-19 pandemic. She was able to explain the origin of this virus, how it spreads, and how one can protect themselves from contracting it. She asserted that:

“So the COVID-19 pandemic I’d say is a virus that uh that originates from China, like that’s the information that we have. Umm it spreads through the droplets from the surfaces, it’s a virus that has uh shows for instances that you have a flue like symptoms and then it’s respiratory if I may say so yah. In order to contain the whole virus you need to uh taking necessary precaution in place by making sure you clean up the bacteria that’s in surfaces, social distancing etc.”

**P3/F/26/PS.**

*Theme 2: The role of the School Management Team (SMT) during COVID-19 pandemic within 4IR context*

The SMT played an important role in ensuring that teaching and learning of the school continues uninterrupted. The teacher indicated that the SMT did not have lot of strategies at hand for introducing online lessons however with time; they were able to come up with strategies that they implemented to continue with the lessons. This was evident in the statement from the interview below:

“We were working so we had to send our lesson plans on the platform called google classroom not sure if you know it. During the lockdown, we were sending lesson plans and videos to the parents. I think the school wasn’t really sure on how they would go about the online learning so I think they took two weeks to plan so during that period we would take a video with your phone and send to parents via email or phone or via the google drive so yah.”

**P3/F/26/PS.**

*Theme 3: The challenges provided by the COVID-19 within the 4IR context of education*

She also indicated that she faced certain challenges during the COVID-19 pandemic, which hindered her from incorporating 4IR in teaching and learning. Load shedding and poor network connection were some of the challenges she encountered. This was evident in the statement from the interview below:

“Okay the bigger challenge I encounter is uh load shedding uh because in our school we obviously have to use Wi-Fi because if there’s electricity we can use Wi-Fi. There was a time where we have to cancel the lessons for the day because we didn’t have electricity so load shedding is the biggest challenge and poor network connection. So with poor network connection you can’t have a lesson.” **P3/F/26/PS.**

*Theme 4: The opportunities provided by COVID-19 within the 4IR context of education*

COVID-19 pandemic brought lot of opportunities in teaching and learning. The teacher was able to incorporate 4IR in her lessons. Numerous platforms for 4IR were implemented as indicated in the statement below:

“Uh I am definitely umm for me teaching in a private school we have to use technology to teach I’d say uh maybe 90% of the time. From the time, umm the pandemic started uh before we even got back to work uh we starting teaching uh our online lessons to the learners uh. How we integrated 4IR uh we had what we call zoom calls or zoom lessons sorry or lessons via google meet so that when the child logs in each learner has email address to login into our lessons, we have codes and my code for example would be math’s. I’d create slides for my lessons and then my slides would appear on the screen so I’d teach along with my slides. Uh another example is sending uh google meet not google meet uh google forms so I found out that was a very alight part coz I get immediate responses as to how learners are grasping the objective.” **P3/F/26/PS.**

It was evident that the COVID-19 pandemic provided the teacher with opportunities to use 4IR platforms such as technology frequently, teach online lessons, zoom lessons, google meet and google forms. This enabled the teacher to have effective lessons, as she was able to assess work given to learners using these 4IR platforms. Furthermore, she indicated that 4IR and the COVID-19 pandemic enabled teachers to bridge the gap between traditional teaching methods and online learning.

### Case 4: Participant 4/Female/25/Government school= P4/F/25/GS

*Theme 1: Understanding of 4IR and coronavirus pandemic (COVID-19) by teachers*

During the interviews that were conducted with the teacher, she demonstrated that she has an idea as to what 4IR was all about. She asserted that 4IR is:

“Uh for me is using Technology to make learning and teaching easier. Bringing the outside world into a classroom environment.” **P4/F/25/GS.**

We had to probe further to understand what she means by using 4IR to bring outside world in the classroom environment. She indicated that:

“Okay for example, during the lesson. Uh let’s take for example in Mathematical Literacy we teach about budgets so there are pupils who have never seen uh for an example an ATM or a bank. So allowing them to see those pictures in a classroom makes it easier for them to relate to the outside world with what they are learning.” **P4/F/25/GS.**

Her explanations led us to enquire what she comprehends about COVID-19. She asserted that:

“Uh COVID-19 is abbreviated from Corona Virus 2019” **P4/F/25/GS.**

*Theme 2: The role of the School Management Team (SMT) during COVID-19 pandemic within 4IR context*

For teaching and learning to be effective, SMT’s need to support teachers in order to achieve that goal. However, that was not the case with this teacher as her SMT was not supportive towards the incorporation of 4IR during COVID-19. In fact, based on our findings it was evident that there was no unity amongst staff members as seen in the statement below:

**“**Eish what I observed is that school management team do not take you seriously. You don’t have a say so they just look at you like hmmm. They judge you because of our age. Hey from what I have experienced with my staffroom. Number 1 there is no teamwork or communication, everyone was doing what they thought was right for themselves and their subject. So there was no form of communication as to what can we use to better the education of all the learners in all subjects, none of them.” **P4/F/25/GS.**

*Theme 3: The challenges provided by COVID-19 within the 4IR context of education*

Lack of support from the SMT was not the only challenge that the teacher experienced especially when she had to incorporate 4IR during the COVID-19 pandemic. We had to find out about her actual teaching experience during COVID-19. She indicated that:

“Ey as a first time teacher I must say that it was the worse the worse experience. I was teaching for the first time last year and there was COVID-19. So I did not enjoy it. Plus we using the environment where we cannot use technology in our classrooms because of lack of resources. There is no electricity in our classes. There are no smart boards so it was a bit difficult.” **P4/F/25/GS.**

*S*he mentioned that lack of resources and lack of electricity in the classroom were major challenges as it hindered the incorporation of 4IR, as the classrooms did not have electrical plugs. She indicated that she did not integrate 4IR during COVID-19. We further probe to find out if she did or did not use some of the 4IR tools such as WhatsApp or Facebook. She asserted that:

“It did not cater for all learners. Not all of them had access to all those things. So even if some of them had access to that, when you reopen you were supposed to repeat what you said so that everyone could be catered for.” **P4/F/25/GS.**

Furthermore, the socio-economic background of the learners served as a challenge as a majority of them came from poor backgrounds. This led us to find out the percentage of learners who have smart phones. She indicated that less than 60% of learners have access to smart phones. We had to find out about the opportunities that 4IR could bring in teaching and learning. She indicated that:

“Hmm okay for teachers it will make our jobs very easier because we will stop using papers and use technology. For learners will mean that when they leave high school they will be computer literate they would have been exposed to technology more than using physical books because the world is moving towards eLearning now. It saves time as well. I believe that if our schools were well resourced it would save us time especially with photocopying and all of that.” **P4/F/25/GS.**

*Theme 4: The opportunities provided by COVID-19 within the 4IR context of education*

In order for the incorporation of 4IR to be success, the teacher suggested that:

“Uh okay so firstly I feel like it’s going to be important for teachers to sit down and communicate properly. Come up with strategy or method that will make it easier for us to teach and be able to finish the content in time. With regards to 4IR, hmm I feel like it’s going to be easier if examples that learners can relate too are used rather than the old way of teaching, because what I picked up is that the textbook is using old examples. So technology makes it easier for us to use new things but it’s going to be difficult because number 1 not all parents can afford to buy cell phones or laptops and I don’t think the school could resource or fund us with electricity so that we can bring laptops in our classrooms. Because at our school there’s only one classroom that has, that can be used with a projector and those learners cannot fit in that classroom.” **P4/F/25/GS.**

## Discussion

### Understanding of the4IR and corona viruspandemic (COVID-19) by teachers

Ramukumba (2019) described 4IR as a third revolution, which combines multiple technologies from the digital, biological and physical worlds. This is so because it provides opportunities for various countries to advance themselves through innovative technology that is driven by growth. It is the advanced technology based on communication and information ( Min *et al.,* 2019). This is similar to **P1/F/46/TS** who indicated that 4IR consist of technology, internet and the use of electronic devices to make teaching and learning easier. A similar perspective was given by **P4/F/25/GS** when she mentioned that 4IR has to do with technology used to enhance teaching and learning.

The Minister of Basic Education emphasised on the need to enhance the quality of teaching and learning in schools in order to prepare for 4IR adequately ( Motshekga, 2018). Hence, it is important for teachers to have access to internet and technology in schools in order to prepare the next generation for the 4IR ( Doucet *et al.*, 2018). By doing so, teachers will be able to share skills such as innovation, critical thinking, and the ability to solve problems and collaboration rather than only focusing on technology and internet access.

The 4IR has brought numerous changes on how learners are taught and how they must learn ( Ilori and Ajagunna, 2020). Hence, **P3/F/26/PS** had a different view regarding her understanding of 4IR where she asserted that 4IR does not reflect the teacher but it is there to make teaching and learning easier. Similar perspectives where also evident when **P2/M/40/TS** described 4IR as a system that is used for teaching and learning to reduce a direct contact between the teacher and a learner. This is similar to Scepanovič (2019) who revealed that 4IR has altered the way we do things.

These assertions reveals that there is still more work that needs to be done in order for teachers to have an in-depth understanding of 4IR. Hence, the President of South Africa said; “the country and teachers needed to change the direction of secondary school education to develop relevant skills to match the fourth industrial revolution” ( Ramaphosa, 2019). This is so because most teachers that taught in the old system are not fully equipped on the use of computers.

For the purpose of this paper, we had to tap into what the teachers understands about the COVID-19 pandemic. A study by Cennimo (2021) defined COVID-19 as an illness that is caused by a novel coronavirus which is now referred to as acute respiratory syndrome coronavirus 2 which was first identified in Wuhan City, Hubei Province, China. This is similar to **P3/F/26/PS, P4/F/25/GS,** and **P2/M/40/TS** who indicated that COVID-19 is a virus that originates from China, which spreads through droplets from the surfaces.

This relates to who revealed that COVID-19 is transmitted through respiratory droplets ( Kord, *et al.,* 2020). There are measures such as disinfecting surfaces, wearing of personal protective equipment, lockdown, and social distancing that can be implement to assist in preventing and controlling the COVID-19 pandemic ( Xiao and Torok, 2020). This is similar to **P1/F/46/TS** who indicated that she adheres to COVID-19 regulations such as using hand sanitisers and maintaining social distancing in class.

### The role of the School Management Team (SMT) during the COVID-19 pandemic within 4IR context

For the purpose of this paper, we had to understand the leadership role that is played by SMT especially during this pandemic. SMT plays an important role in giving leadership guidance, assistance and direction in teaching and learning situation ( Mathipa *et al.,* 2014). This is similar to **P3/F/26/PS** who indicated that the SMT did manage to provide teachers with support needed for teaching and learning even though they encountered challenges. This showed that the SMT managed to improve the quality of teaching and learning as they were able to assist teachers to continue with teaching and learning during the pandemic ( Kgothe, 2013).

However, that was not the case with **P1/F/46/TS, P2/M/40/TS,** and **P4/F/25/GS** as there was no support or guidance from the SMT. This is similar to **P3/F/26/PS** who revealed that most SMT members have a challenge when they have to work as a team and some members do not know their roles in the SMT ( Maja, 2016). As a result, this affects the management effectiveness, impacts negatively on the leadership and affect teaching and teaching.

### The challenges provided by the COVID-19 within the 4IR context of education

Teachers encountered numerous challenges during COVID-19 pandemic when they had to integrate 4IR during teaching and learning process. A study conducted by Kayembe and Nel (2019) revealed that lack of funding, resources and skills are major challenges that hinders the incorporation of 4IR. Connectivity is an important aspect towards successful integration of 4IR in teaching and learning but most of public schools do not have access to internet ( Moyo, 2019).

This is similar to **P1/F/46/TS, P3/F/26/PS, P2/ F/46/TS,** and **P4/F/25/GS** as they mentioned that lack of teaching and learning resources, access to internet, and socio-economic background are some of the challenges that hindered the incorporation of 4IR effectively. Furthermore, Oke and Fernandes (2020) revealed that there is insufficient knowledge regarding the acceptability and consequences of the 4IR in the education sector. This is similar to **P1/F/46/TS** who indicated that inadequate knowledge and skills of using technology were some of the factors that hindered 4IR incorporation. Hence, Feza (2019) indicated that the incorporation of 4IR and advanced technology in teaching and learning would be a challenge on teachers who are already been overworked.

### The opportunities provided by the COVID-19 within the 4IR context of education.

The incorporation of 4IR in teaching and learning process has developed many opportunities for teachers, such as the use of google in classroom situation, which has replaced the teacher as the oracle of knowledge ( Naude, 2019). This is similar to **P3/F/26/PS** who indicated that Google meet were one of the tools that contributed successfully in the integration of 4IR during the COVID-19 pandemic. This clearly indicates that 4IR has opened up new opportunities that allows new and innovative solutions to problems ( Munasi and Madikizela-Madiya, 2020). This is so because **P1/F/46/TS, P2/M/40/TS, P3/F/26/PS,** and **P4/F/25/GS** indicated that 4IR tools such as smartboards, Google meets, WhatsApp, Zoom, YouTube, Microsoft Teams and Zoom were used to integrate 4IR during the COVID-19 pandemic. Hence, it is important for schools to be equipped for teaching with and 4IR ( Musgrave, 2019).

Therefore, it is recommended that the government should develop interventions in training of the teachers for professional development in the usage of 4IR for teaching and learning activities. These will enable the teachers to be more skillful and competent in the implementation of 4IR technology. Furthermore, adequate support is also required from the government and stakeholders in the education sector concerning the supply of resources such as smartboards, computers, and network access in schools lacking such facilities. The limitations of this paper is that the sample was small but the advantage is that the findings can be inferred to similar cases. Only one data collection technique was used.

## Conclusion

In responding to the following research questions; How does the teacher understanding of 4IR and COVID-19 shape the integration of 4IR in teaching and learning?; What was the role of school management teams in the integration of 4IR in teaching and learning during COVID-19 pandemic?; What are the challenges and opportunities provided by the 4IR during the COVID-19 within the context of education? The findings showed that there is lack of teaching and learning resources, access to internet and socio-economic background, which were some of the challenges, hindered the integration of 4IR in the teaching and learning process. There were also challenges related to teachers’ background on the usage of 4IR and lack of SMT support during COVID-19. It can be inferered from the findings that the integration of 4IR in the teaching and learning was negatively affected by the existence of the above-mentioned challenges, which we suggest that they need to be addressed.

## Data Availability

Figshare: CASE 1 4IR TRANSCRIBED DATA.docx. https://doi.org/10.6084/m9.figshare.22664680.v2 ( Mudau, 2023a). The project contains the following underlying data:
• CASE 1 4IR TRANSCRIBED DATA.docx. (Anonymised transcription of interview with participant 1 - P1/F/46/TS). • CASE 2 4IR TRANSCRIBED DATA.docx. (Anonymised transcription of interview with participant 2 - P2/M/40/TS). • CASE 3 4IR TRANSCRIBED DATA.docx. (Anonymised transcription of interview with participant 3 - P3/F/26/PS). Data are available under the terms of the Creative Commons Attribution 4.0 International license (CC-BY 4.0). Figshare: Case 4. https://doi.org/10.6084/m9.figshare.22775678.v1. ( Mudau, 2023d).
• CASE 4 4IR TRANSCRIBED DATA.docx. (Anonymised transcription of interview with participant 3 - P4/F/25/GS) Data are available under the terms of the Creative Commons Attribution 4.0 International license (CC-BY 4.0). Figshare: interview schedule. https://doi.org/10.6084/m9.figshare.22664740.v1 ( Mudau, 2023b). This project contains the following extended data:
• Interview schedule.pdf. (Interview questions used in this study). Figshare: SRQR checklist for ‘Technology integration during teaching and learning: the COVID-19’. https://doi.org/10.6084/m9.figshare.22664818.v1 ( Mudau, 2023c). Data are available under the terms of the Creative Commons Attribution 4.0 International license (CC-BY 4.0).
