# Supplementary material for: The influence of the fourth industrial revolution in teaching and learning: the COVID-19 context
Source: F1000Res. 2025 Apr 11;12:1183. Originally published 2023 Sep 21. [Version 3] doi: 10.12688/f1000research.131618.3 (PMC12120419; doi:10.12688/f1000research.131618.3)
Supplement: Peer review history

# Reviewer report: Reviewer response for version 3

**DOI:** 10.5256/f1000research.180379.r378308

**Published:** 2025-05-29

**Authors:** Samuel Ouma Oyoo

**Affiliations:** 1 Maseno University, Maseno, Kisumu, Kenya

I have reviewed an earlier version of this paper and made suggestions for the attention of the authors. The current version is a much better version as it has addressed all my concerns as expressed to the authors in my earlier submission. This paper comprehensively covers the adoption of technology in science teaching and learning in South African schools though anchored on COVID-19 circumstances. It is a sound contribution to both practice and policy on integration of technology in teaching and learning of (physical) sciences in not only South Africa but also in similar contexts.

Is the work clearly and accurately presented and does it cite the current literature?

Yes

If applicable, is the statistical analysis and its interpretation appropriate?

Not applicable

Are all the source data underlying the results available to ensure full reproducibility?

Yes

Is the study design appropriate and is the work technically sound?

Yes

Are the conclusions drawn adequately supported by the results?

Yes

Are sufficient details of methods and analysis provided to allow replication by others?

Yes

Reviewer Expertise:

Science Education, Science Teacher Education, Science teacher’s classroom practice, language and technology in in science pedagogy.

I confirm that I have read this submission and believe that I have an appropriate level of expertise to confirm that it is of an acceptable scientific standard.

# Reviewer report: Reviewer response for version 3

**DOI:** 10.5256/f1000research.180379.r377368

**Published:** 2025-04-12

**Authors:** Layane Thomas Mabasa

**Affiliations:** 1 University of Limpopo, Mankweng, Limpopo, South Africa

The authors did attempt to respond to the concerns raised. However, they did not address all of them. For example, details on ethical issues and the explanation of the multiple case study design have not been addressed.

Is the work clearly and accurately presented and does it cite the current literature?

No

If applicable, is the statistical analysis and its interpretation appropriate?

Not applicable

Are all the source data underlying the results available to ensure full reproducibility?

No source data required

Is the study design appropriate and is the work technically sound?

No

Are the conclusions drawn adequately supported by the results?

Partly

Are sufficient details of methods and analysis provided to allow replication by others?

No

Reviewer Expertise:

My areas of expertise are in  research methodology, Educational leadership and governance

I confirm that I have read this submission and believe that I have an appropriate level of expertise to confirm that it is of an acceptable scientific standard, however I have significant reservations, as outlined above.

# Reviewer report: Reviewer response for version 2

**DOI:** 10.5256/f1000research.168211.r296347

**Published:** 2024-07-26

**Authors:** Samuel Ouma Oyoo

**Affiliations:** 1 Maseno University, Maseno, Kisumu, Kenya

This was a revised version of the version I had reviewed and shared my comments on earlier. I am satisfied that the author(s) has attended to all my concerns in total.

Is the work clearly and accurately presented and does it cite the current literature?

Yes

If applicable, is the statistical analysis and its interpretation appropriate?

Not applicable

Are all the source data underlying the results available to ensure full reproducibility?

Yes

Is the study design appropriate and is the work technically sound?

Partly

Are the conclusions drawn adequately supported by the results?

Yes

Are sufficient details of methods and analysis provided to allow replication by others?

Yes

Reviewer Expertise:

Science Education, Science Teacher Education, Science teacher’s classroom practice

I confirm that I have read this submission and believe that I have an appropriate level of expertise to confirm that it is of an acceptable scientific standard.

# Reviewer report: Reviewer response for version 2

**DOI:** 10.5256/f1000research.168211.r296348

**Published:** 2024-07-08

**Authors:** Layane Thomas Mabasa

**Affiliations:** 1 University of Limpopo, Mankweng, Limpopo, South Africa

On the second version of the manuscript, the report is that the authors addressed some of the concerns but not all. The concerns that have not been addressed include the anonymisation of the name of the institution that issued the Ethics certificate, ethical issues as advised in the earlier report, clarity on the kind of case study used, for example, how is this a multiple case study, and clarity on whether teachers are cases or participants. The reference by Moyo in the list of references be moved to an appropriate space to follow the list alphabetically. Consequently, the status remains the same which is **"Approved with reservations".**

 Kind regards,

 LT Mabasa

Is the work clearly and accurately presented and does it cite the current literature?

No

If applicable, is the statistical analysis and its interpretation appropriate?

Not applicable

Are all the source data underlying the results available to ensure full reproducibility?

No source data required

Is the study design appropriate and is the work technically sound?

No

Are the conclusions drawn adequately supported by the results?

Partly

Are sufficient details of methods and analysis provided to allow replication by others?

No

Reviewer Expertise:

My areas of expertise are in  research methodology, Educational leadership and governance

I confirm that I have read this submission and believe that I have an appropriate level of expertise to state that I do not consider it to be of an acceptable scientific standard, for reasons outlined above.

# Reviewer report: Reviewer response for version 1

**DOI:** 10.5256/f1000research.144479.r237897

**Published:** 2024-02-28

**Authors:** Samuel Ouma Oyoo

**Affiliations:** 1 Maseno University, Maseno, Kisumu, Kenya

The study reported here is a worthy contribution to the field of science education, especially with regard to curriculum delivery in the context of the study. This included issues on the role of the school management teams and at different levels of schooling in the context of the study. The place of use of technology in teaching as may have been made more urgent by the prevalence of covid-19 came out clearly. These are issues that will now be applicable to continued delivery of science content in the schools in the context of the study. 

 Reading through the work however, the authors needed to give closer attention to the grammar issues including the tenses. There are instances where the work should have been in the past tense - covid-19 is no longer a challenge in the context of the study and the writing should be to address that fact. 

 While the design including the data analysis approach is appropriate, the results are reported with the apparent assumption that all the readers will be well versed in the education system in the context of the study. The authors need to brief up on the system of education in the context of the study including issues on the types of schools and how they are relatively endowed. The participants were drawn from the respective types/categories and levels of schooling in the context of the study.   The results will thereafter relate better to the study context. 

 The authors need to represent the participants and their respective locations more accurately. Two of the participants are reported to have moved to Gauteng from Limpopo province yet there is still mention of them as having been in Limpopo at the time of the study. This will make it clear that the study was conducted in two provinces (Gauteng and Mpumalanga), not three (Gauteng, Limpopo and Mpumalanga). 

 The conclusion is well presented. However, the paragraph on recommendations that comes immediately above the conclusion can be moved to become the very last paragraph of the work.

Is the work clearly and accurately presented and does it cite the current literature?

Yes

If applicable, is the statistical analysis and its interpretation appropriate?

Not applicable

Are all the source data underlying the results available to ensure full reproducibility?

Yes

Is the study design appropriate and is the work technically sound?

Partly

Are the conclusions drawn adequately supported by the results?

Yes

Are sufficient details of methods and analysis provided to allow replication by others?

Yes

Reviewer Expertise:

Science and pedagogy: Current focui are in (1) School science teachers classroom practice including use of technology in the transformation of science content (2) Language use icluding teacher talk for and in teaching science.

I confirm that I have read this submission and believe that I have an appropriate level of expertise to confirm that it is of an acceptable scientific standard, however I have significant reservations, as outlined above.

# Response

**Published:** 2024-06-20

**Authors:** Awelani Mudau

**Affiliations:** Science and Technology Educati, University of South Africa, Pretoria, Gauteng, South Africa

We are grateful for your comments. They have been addressed

# Reviewer report: Reviewer response for version 1

**DOI:** 10.5256/f1000research.144479.r226456

**Published:** 2024-02-14

**Authors:** Layane Thomas Mabasa

**Affiliations:** 1 University of Limpopo, Mankweng, Limpopo, South Africa

Reviewer Report

 F1000 Research Journal

 Title: Technology integration during teaching and learning: the COVID-19 context

1. **Introduction**

This report is composed of the different aspects that are necessary for decision-making regarding the readiness of the manuscript to be considered for approval  by the F1000 Research Journal. It outlines the introduction, the strong points, the weaknesses, and the approval status.

1. **Strong points of the manuscript**

Even though the manuscript has several weaknesses which will be outlined later, it has strong points. The strong points are that:

 The abstract is in line with the journal's expectations.

 There is consistency in terms of how sources have been referenced.

 There is an attempt by the authors to use current literature.

 Verbatim quotations of the responses from the participants have been used to give voice to the participants.

 The manuscript is within the scope as it focuses on technology integration during teaching and learning in the context of the COVID-19 pandemic.

  

1. **Weak points of the manuscript**

Some weaknesses need to be addressed to improve its quality and by the F1000 Research Journal. Weaknesses are that:

 The title seems to suggest that the study is about technology integration during teaching and learning, whereas in the text, the focus is on the Fourth Industrial Revolution (4IR). The question that arises will be “Is technology integration during teaching and learning the same as the Fourth Industrial Revolution (4IR)?” The authors need to explain and align the title accordingly. 

 The claim made in par.2, sentence no.1 under **Introduction **needs to be supported by a source.

 The manuscript can benefit from another editing phase. For example, the spelling of “South Arica” in par.2, sentence no.1 under **Fourth Industrial Revolution (4IR)** needs to be written as “South Africa”. There is a need to use past tense in sentence no.1, par.1 under **Effects of COVID-19 pandemic in the teaching and learning process. **The word “affects”, needs to be written as “affected”. The last but one sentence under the **Blended learning approach during the COVID-19 pandemic **needs to be edited. Sentence no.2 in par.2, under **Theoretical framework,** is not clear. It needs to be rephrased. Sentence no.1 in par.4 under **Theoretical framework** is not clear. It needs to be rephrased. There is a need to edit sentence no.2 in par.2 under **The role of the School Management Team during the COVID-19 pandemic with 4IR context.**

 The authors under **Research Design and Methodology**, indicate that the manuscript is based adopted a multiple case study design for use in the study. However, there is no indication of how the study is a multiple-case study. The four teachers' participation does not mean that this is a multiple-case study. The question that arises is what is the case in this study? The participants in this context are not cases. The case seems to be the integration of technology during teaching and learning and not the teachers. The authors may also improve this section by indicating how the constructivism theory guided them to adopt the semi-structured interviewing tool for use to generate data. They can also indicate settings where interviews were held, challenges, and how they dealt with the challenges.

 There is a need to explain ethical issues in detail. The authors may add respect, dignity and care, benefits, risks, and harm, privacy and anonymity, and how data were managed and protected. There is a need to anonymise the university that issued the ethics certificate under the **Ethical statement**.

 Due to the nature of the study, which is qualitative, there is a need for authors to use concepts that are in line with qualitative studies. For example, instead of using the concept “validity”, authors may use “trustworthiness” which can be enhanced through credibility, confirmability, transferability, and dependability.

 There is a need for a detailed explanation of the way in which data were analysed and themes developed.

 The themes listed above the sub-heading on results are four but those listed under **Results** are three. Is there any justification for the omission of the fourth theme **Results**?

 Authors are also advised to avoid using gender related designations like “he” or “she”. This can be done by using codes developed in line with anonymity. Furthermore, instead of discussing the responses of the participants separately, all their responses can be discussed under one theme. For example, the theme on Understanding of 4IR and coronavirus pandemic (COVID-19) by teachers, authors may indicate the responses by P1/F/46/TS, P2/M/40/TS, P3/F/26/PS and P4/F/25/GS. Where their understanding is the same, the authors may indicate that, and where their understanding is different the authors may indicate that and give an interpretation of their similarities and or differences.  In its current form, the manuscript is difficult to read and makes it a bit too long.

 There is a need to rewrite the **Conclusion **section of the manuscript. This can be done by indicating how research questions were answered and the aim and objectives of the study were achieved through the results and the findings of the study. There is also a need to highlight the contribution of the study to the knowledge base.

 On the list of references, a source by Moyo (2019) may be removed where it is currently placed, and be placed in an appropriate slot following the alphabetical order as it has been done with the other sources.

1. **The approval status**

Noting that the manuscript has some strong points, it can be considered after major issues and concerns have been addressed. This is because some of the concerns raised under the weaknesses section, do affect the conceptualisation of the study.  I therefore recommend Approved with reservations. More significant revisions need to be done.

Is the work clearly and accurately presented and does it cite the current literature?

No

If applicable, is the statistical analysis and its interpretation appropriate?

Not applicable

Are all the source data underlying the results available to ensure full reproducibility?

No source data required

Is the study design appropriate and is the work technically sound?

No

Are the conclusions drawn adequately supported by the results?

Partly

Are sufficient details of methods and analysis provided to allow replication by others?

No

Reviewer Expertise:

My areas of expertise are in  research methodology, Educational leadership and governance

I confirm that I have read this submission and believe that I have an appropriate level of expertise to confirm that it is of an acceptable scientific standard, however I have significant reservations, as outlined above.

# Response

**Published:** 2024-06-20

**Authors:** Awelani Mudau

**Affiliations:** Science and Technology Educati, University of South Africa, Pretoria, Gauteng, South Africa

We are grateful for the comments. They were plausible indeed and they should make our paper better.
